# Uninformative Biological Variability Elimination in Apple Soluble Solids Content Inspection by Using Fourier Transform Near-Infrared Spectroscopy Combined with Multivariate Analysis and Wavelength Selection Algorithm

**DOI:** 10.1155/2017/2525147

**Published:** 2017-10-16

**Authors:** Lin Zhang, Baohua Zhang, Jun Zhou, Baoxing Gu, Guangzhao Tian

**Affiliations:** College of Engineering, Nanjing Agricultural University, Nanjing, Jiangsu 210031, China

## Abstract

Uninformative biological variability elimination methods were studied in the near-infrared calibration model for predicting the soluble solids content of apples. Four different preprocessing methods, namely, Savitzky-Golay smoothing, multiplicative scatter correction, standard normal variate, and mean normalization, as well as their combinations were conducted on raw Fourier transform near-infrared spectra to eliminate the uninformative biological variability. Subsequently, robust calibration models were established by using partial least squares regression analysis and wavelength selection algorithms. Results indicated that the partial least squares calibration models with characteristic variables selected by CARS method coupled with preprocessing of Savitzky-Golay smoothing and multiplicative scatter correction had a considerable potential for predicting apple soluble solids content regardless of the biological variability.

## 1. Introduction

Soluble solids content (SSC) is a major internal attribute of apples which mostly determines fruit flavor, harvest time, and postharvest storage requirements [1]. High SSC has been associated with optimal fruit maturity and high consumer preference. Consequently, it is of great significance to develop methods for nondestructive, rapid, and reliable measurement of apple SSC to meet growing consumer requirements for high quality fruit.

Near-infrared (NIR) spectroscopy is a rapid, accurate, and nondestructed inspection technique used in the fruit industry for predicting the optimal picking date, reducing losses during storage, and classifying fruit into two quality grades based on quality index [1–4]. Combined with various statistical methods, such as multiple linear regression (MLR), principle component regression (PCR), and particle least squares (PLS), NIR spectroscopy can be used to establish effective prediction models for the composition or properties of unknown samples. The models have been successfully applied to measure a wide range of apple nutritional value indices and internal quality properties such as firmness, pH, acidity, and especially the SSC [2, 5, 6]. In addition, direct relationship between various Vis/NIR wavelengths and sensory attributes of apples has been confirmed [7, 8]. There are already some complete commercial solutions available to in-line and on-site complete measurement of fruit quality attributes, yet their accuracy and robustness are still worth improving [9].

The validity and reliability of the calibration models for future predictions depend on how well the calibration set represents the composition of new samples [8]. The difference of internal structure and composition of apples influenced by their external physical properties (including size, shape, color, texture, and temperature) and biological properties (including cultivar, season, maturity level, and geographical origin) leads to the changes in the spectrum, which in turn is the basis for internal quality measuring [8, 10, 11]. However, a great variety of physical and biological properties also influence the optical propagation properties and interaction behaviors with incident light, thus decreasing the external and internal quality inspection accuracy [12]. Compensation models for eliminating the spectral variation caused by fruit physical variability and biological variability have been built [9, 10, 13, 14]. Nevertheless, most of the researches have focused on updating the calibration models with more new variability in the calibration set or establishing specific models for different conditions, the former of which may simultaneously increase the complexity and decrease the accuracy of the calibration model, while for the latter, it may be difficult to estimate the individual variability [7, 9, 12]. Moreover, many attempts on wavelength selection, spectra correction, and instrument noise reduction show good market prospects [3, 15–17]. In addition to information about the samples, NIR spectra also contain background noise; thus it is crucial to correct for the nonlinearity by mathematical signal treatments. Furthermore, the problem of multicollinearity among contiguous variables makes wavelength selection necessary, which can improve model performance and robustness by identifying and removing useless, noisy, and redundant variables. Key wavelengths selection saves overall time for the analysis, making the model more suited for automated industrial applications. In order to develop robust models to predict the SSC of apples, elimination methods of uninformative variability in calibration model correction combined with multivariate regression analysis and wavelength selection algorithm should be investigated.

## 2. Objectives

The main objective of this paper was to investigate the multivariate regression analysis combined with preprocessing methods and wavelength selection algorithms for eliminating uninformative biological variability in apple SSC calibration models and to develop robust models for determining SSC in apples. Several subobjectives have to be fulfilled in order to achieve the above-mentioned objective: (1) collecting the spectral data of apple samples in the region of 14,000 to 3,800 cm^−1^ by using the Antaris II FT-NIR spectrometer; (2) developing the full-spectrum (4000–10000 cm^−1^) PLS models for apple SSC prediction using raw spectra; (3) introducing preprocessing methods to eliminate the uninformative biological variability and conducting full-spectrum PLS models based on pretreated spectra; (4) picking out effective variables from original data to simplify the computational complexity using wavelength selection methods and establishing calibration models based on optimal variables; (5) verifying and evaluating the performance of calibration models based on the correlation coefficient of calibration (*R*_*C*_^2^) and that of cross-validation (*R*_*p*_^2^), as well as the root mean square errors for calibration (RMSEC), cross-validation (RMSECV), and prediction (RMSEP).

## 3. Materials and Methods

### 3.1. Apple Samples

A total of 160 “Fuji” apples free of physiological decay or rot and physical damage (e.g., bruises and cuts) were purchased from an orchard in Shandong province, China. All samples were individually washed, dried, numbered, and then marked sampling point around the equator. The samples were stored in laboratory (temperature, 20°C; relative humidity, 60%) for 12 h before experiment to allow the samples to reach room temperature to reduce the effect of apple temperature on the prediction accuracy [18].

### 3.2. FT-NIR Spectra Collection

The FT-NIR spectra of apples were collected in the diffuse reflectance mode using an Antaris II FT-NIR spectrometer (Thermo Scientific Co., USA) equipped with an integrating sphere, a NIR fiber-optic probe, a high sensitivity InGaAs detector, and a tungsten lamp (20 W). Each spectrum was the average of 32 scans and was recorded as absorbance value (log⁡(1/*R*)), where *R* = reflectance. The data was collected with 8.0 cm^−1^ spectral resolutions in the spectral range of 14,000 to 3,800 cm^−1^. The distance between the fiber and apple was ≤5 mm while measuring. In order to avoid surface reflectance and guarantee subsurface penetration of the light into the apple flesh, the fiber-optic probe was placed on 75-degree inclines [19]. The calibrated reflectance was calculated as the percentage of the reflection of a standard reference material (BaSO_4_, of 99% reflectance, Nicolet Inc.). When the spectrum is collected, each sample was rotated for 3 times by 120° and scanned on the three equidistant positions around the equator. And the average spectrum was taken as the original spectrum of the sample for the following analysis. The temperature was kept around 25°C at a steady humidity level in the laboratory.

### 3.3. Reference Measurements

The SSC values were determined by traditional destructive measurement immediately after spectra acquisition. Three pieces of flesh (1-2 cm) with peel from equidistant points along the equator of each apple at the location of the NIR measurement were filtered through the gauze [13, 14]. Juice from each tissue sample was squeezed and dropped onto a temperature-compensated digital refractometer (ARIAS 500, Reichert Technologies, New York, USA) to measure the actual SSC value. Each sample was measured in two replications using reference method 920.151 of the Official Methods of Analysis (AOAC, 1997) and the average was taken as the SSC value. The refractive index accuracy is ±0.03%, and the °Brix (%) range was 0–95%.

### 3.4. Multivariate Regression Analysis

Partial least squares (PLS) regression is a widely used multivariate statistical technique for the calibration model in NIR analysis. The first step in PLS regression is to decompose the matrix, and the model is given:

(1) X=TP+E,

(2) Y=UQ+F.

The second step is that *T* and *U* are processed by linear regression, which must build the following linear correlation:

(3) $$\begin{array}{c} U=BT+E. \end{array}$$

In these equations, *T* and *U* are the score matrices of *X* matrix and *Y* matrix, *P* and *Q* are the loading matrices of *X* matrix and *Y* matrix, *E* and *F* are the errors that come from the process of PLS regression, and *B* represents the internal relations between *U* and *T*. In order to reach this object, the coordinate of *T* is rotated.

Maximum information from a large number of highly correlated and collinear original *X* variables can be extracted and condensed onto no more than 20 underlying variables called latent variables (LVs) [20]. The LVs were applied as new eigenvectors in the calibration and validation steps to explain the variance and reduce the dimensionality of the original spectra [14]. K-fold cross-validation is commonly applied in LVs determination. In general, cross-validation combines (averages) measures of fit (prediction error) to derive a more accurate estimate of model prediction performance and reduce overfitting problems. In this work, PLS regression was used to develop a quantitative relationship between the spectral data and the apple SSC attributes, of which the optimal number of LVs was selected by minimizing the RMSECV based on 10-fold cross-validation of calibration set.

Samples division into calibration and prediction sets is critical to PLS regression modeling. The Kennard-Stone (KS) algorithm is often applied to select a representative subset from a pool of *N* samples when there is no standard experimental design. In order to divide the objects evenly throughout the descriptor space of the original data set, the KS algorithm firstly takes two samples that are the farthest pair in terms of *x*-vectors Euclidean distance and then it sequentially finds a new object that has the maximum Euclidean distance from the already selected ones. Such a process is repeated until a desired number of samples have been placed to the calibration set, and the remaining samples were then added to the prediction set. The Euclidean distance between *x*-vectors of sample *p* and sample *q* is defined as [21]

(4) dxp,q=xp−xq=∑j=1Nxpj−xqj2p,q∈1,M.

In (1), *N* is the number of variables in *x* and *M* is the number of samples; *x*_*p*_(*j*) and *x*_*q*_(*j*) are the *j*th variable for samples *p* and *q*, respectively.

### 3.5. Solutions for Uninformative Biological Variability Elimination

In order to eliminate the effects caused by uninformative biological variability, various preprocessing methods were employed in the NIR models developed based on PLS regression. Multiplicative scatter correction (MSC) is a transformation technique compensating for the pure addition and multiplication effects in spectral data based on the average spectrum in the data set, while standard normal variate (SNV) removes scatter effects by centering and scaling each individual spectrum (i.e., sample-oriented standardization). Both methods are row-oriented transformations. Savitzky-Golay (S-G) smoothing is one of the most commonly used methods to eliminate noise in the spectral data, especially for filtering high frequency noise, without reducing the number of spectral variables. And a mean normalization (MeanN) step applied prior to PLS regression has been found to be effective to decrease spectral uninformative biological variability due to curvature. These spectral pretreatments were performed in the Unscrambler X10.4 (CAMO PROCESS AS, Oslo, Norway).

### 3.6. Characteristic Wavelength Selection Methods

Characteristic wavelength selection in multivariate regression analysis is crucial to the development of calibration models and can improve the prediction performance and facilitate results interpretation. In this study, competitive adaptive reweighted sampling (CARS) and random frog (RF) algorithm were used for the calibration models to pick out the most effective wavelengths for the SSC determination.

**Figure 1:**
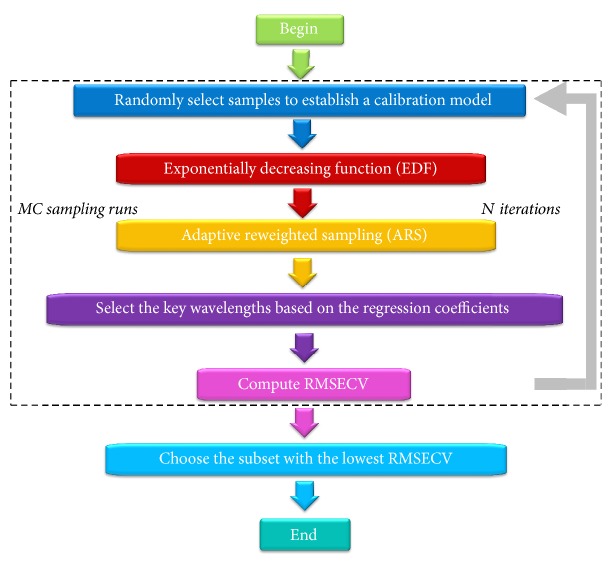
The flowchart of the competitive adaptive reweighted sampling algorithm.

Competitive adaptive reweighted sampling (CARS) is a novel wavelength selection algorithm employing the “survival of the fittest” principle from Darwin's Evolution Theory, and it has been successfully applied to the prediction of SSC and dry matter of pears [11, 22]. CARS method selects *N* wavelength subsets sequentially from the *N* sampling runs in an iterative manner. In each sampling run, a fixed scale sample is first randomly selected to establish a calibration model. Then CARS works in four successive steps: Monte Carlo (MC) model sampling, enforced wavelength reduction by exponentially decreasing function (EDF), competitive wavelength reduction by adaptive reweighted sampling (ARS), and RMSECV calculation for each subset. Finally the subset with the lowest RMSECV value was determined as the optimal subset.  Figure 1 shows the scheme of the CARS algorithm. The key wavelengths selected by CARS are considered as the wavelengths with the large absolute regression coefficients in a multivariate linear regression model. The exponential decay function is used to control the retention rate of variable in the algorithm, and it has the potential to select an optimal combination of the wavelengths [23, 24].

**Figure 2:**
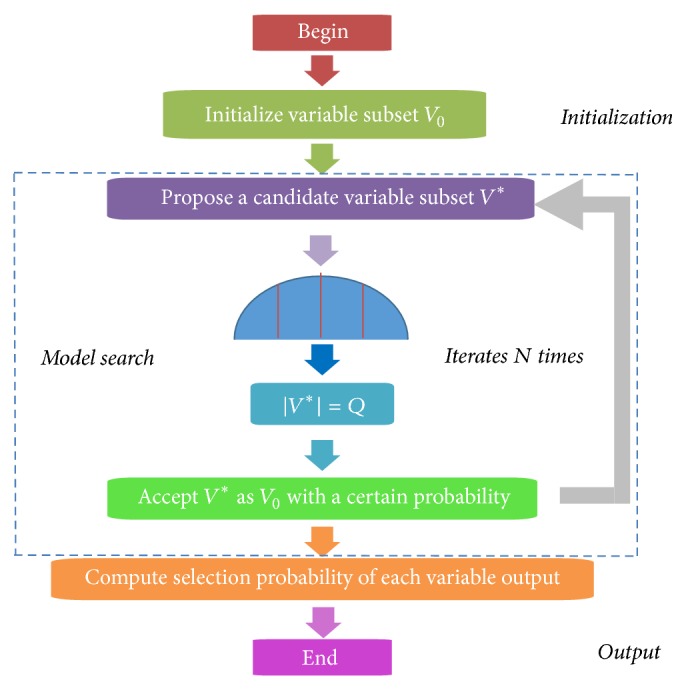
The flowchart of the random frog algorithm.

Random frog (RF) is a useful variables selection technique based on the reversible jump Markov chain Monte Carlo (RJMCMC). Interval random frog was successfully developed and validated for near-infrared spectra [25]. Similar to CARS, it works in iterative way; meanwhile, it calculates the selection probability (SP) for each variable. Characteristic variables are selected according to the SP rank of all variables. The RF operates in the following procedures. (1) Given an initial variable subset *V*_0_ with its cardinality denoted by |*V*_0_| = *Q*, a random number is generated from the normal distribution with mean *Q* and standard deviation *θQ*; this random number is then rounded to its nearest integer, denoted by *Q*^*∗*^. (2) Based on *V*_0_, a candidate variable subset *V*^*∗*^ that contains *Q*^*∗*^ variables is generated; accept *V*^*∗*^ as *V*_1_ with a certain probability and let *V*_0_ = *V*_1_; repeat the above procedures until *N* iterations are finished. (3) Compute a selection probability of each variable and then use it as a criterion for selecting variables. The key steps of RF are illustrated in Figure 2. The advantage of random frog is that no demanding mathematical formulation is needed and no prior distributions need to be specified like in formal RJMCMC methods, which makes it easier to implement and computationally very efficient [26]. There were five tuning parameters controlling the performance of RF which could be optimized in the routines and the most important two parameters among them were the number of iterations *N* and the number of variables contained in the initialized variable set *Q* [26, 27].

### 3.7. Performance Evaluation of the Models

The performance of calibration model was evaluated based on the correlation coefficient of calibration (*R*_*C*_^2^), that of cross-validation (*R*_CV_^2^), and that of prediction (*R*_*P*_^2^), as well as the root mean square errors for calibration (RMSEC), cross-validation (RMSECV), and prediction (RMSEP) [10]. The main evaluation indices for performance in our study were *R*_*p*_^2^ and RMSEP. In addition, the bias was taken into consideration for distinguishing systematic error. Generally, good models should have high *R*_*P*_^2^ but low RMSEP.

## 4. Results and Discussion

### 4.1. Statistics of Measured Parameters

**Figure 3:**
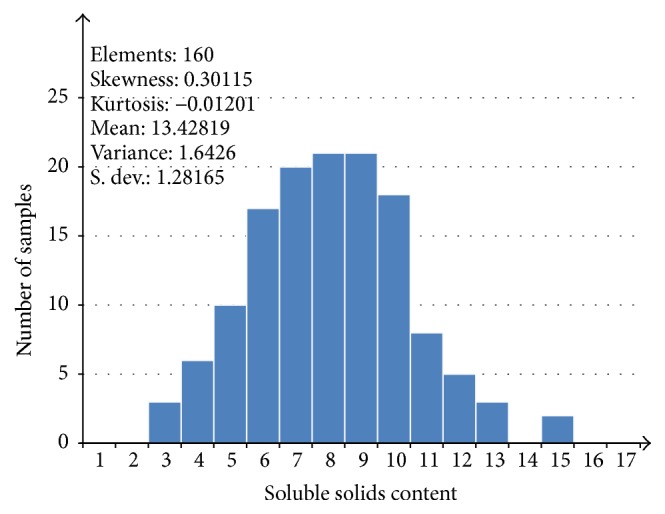
Distribution of soluble solids content of all 160 fruit samples.

SSC measurements for all 160 samples ranged from 10.81% to 17.13%, with the mean, median, and standard deviation of the SSC values of 13.42%, 13.45%, and 1.28%, respectively.  Figure 3 gives the distribution of the spectral data, which was approximately subordinate to the normal distribution. The 160 samples were divided into a calibration set (120 samples) and a prediction set (40 samples) based on Kennard-Stone (KS) algorithm. The descriptive statistics for reference measurements of SSC are presented in Table 1. As seen from Table 1, the range of SSC values in the calibration set covered that of the prediction set, which is helpful when establishing a stable and robust calibration model [28]. Further, there was no significant difference between the standard deviation of the calibration set and that of the prediction set. Therefore, the distribution of the samples is applicable in both the calibration and prediction sets.

### 4.2. Uninformative Biological Variability Elimination

**Figure 4:**
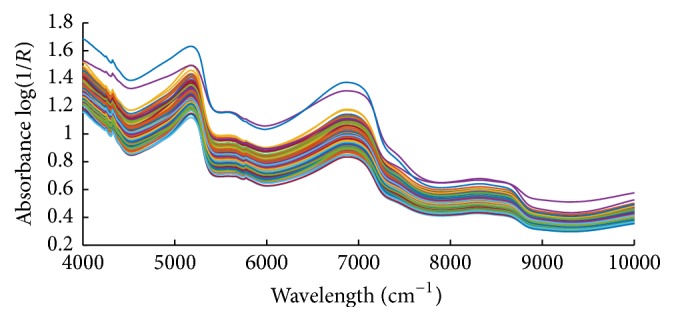
Typical absorbance spectra of all apple samples between 4000 and 10,000 cm^−1^.

**Figure 5:**
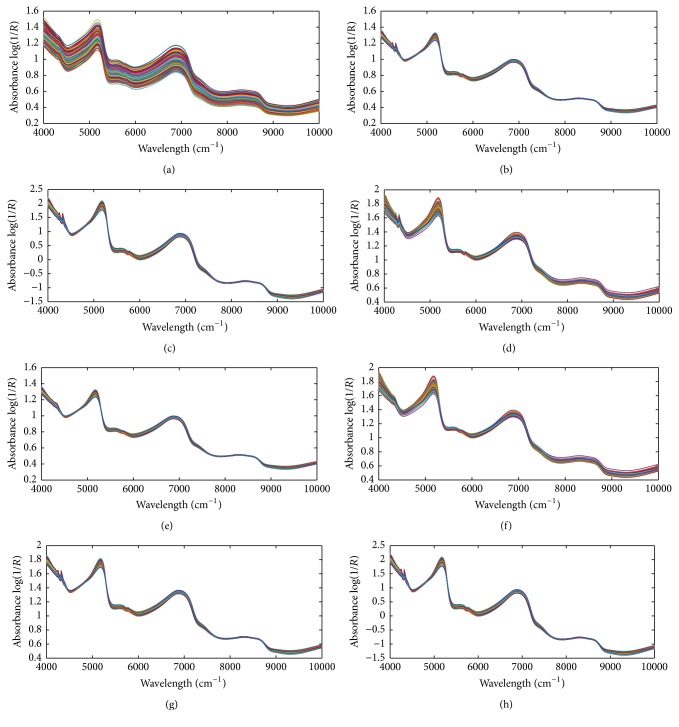
The preprocessed spectra by S-G smoothing (a), MSC (b), SNV (c), MeanN (d), combination of S-G smoothing and MSC (e), combination of S-G smoothing and MeanN (f), combination of MSC and MeanN (g), and combination of MSC and SNV (h).

Since the spectral data below 10,000 cm^−1^ and above 4000 cm^−1^ contained significant noise, a total of 3112 wavelength points between 10,000 cm^−1^ and 4000 cm^−1^ were used in this study.  Figure 4 shows the original FT-NIR spectra of 160 “Fuji” apples samples. Baseline shifts and noises due to light scattering or concentration variations in samples were observed in the spectra with a broad wavelength region. Therefore, it is necessary to preprocess the original spectra to remove irrelevant information which cannot be handled properly by the regression techniques. The pretreated spectra by Savitzky-Golay (S-G) smoothing (39-point), multiplicative scatter correction (MSC), standard normal variate (SNV), and mean normalization (MeanN), as well as their combinations, are shown in Figures 5(a)–5(h), respectively. The spectra pretreated by S-G smoothing in Figure 5(a) were smoothed. While smoothing obviously improved the visual aspect of the raw spectra, it also removes information that cannot be determined to be useless. The pretreated spectra in Figures 5(b) and 5(c) removed the baseline shift, but they also left considerable noise. In Figure 5(d), the features in the region less than 5000 cm^−1^ were enhanced. And the combinations of any two of them also showed good effects in Figures 5(e)–5(h). All of these pretreated spectra were used in the SSC prediction for eliminating the effects caused by uninformative biological variability. Their performances were compared in PLS regression calibration step.

### 4.3. Models for Raw and Processed Full-Range Spectra

**Figure 6:**
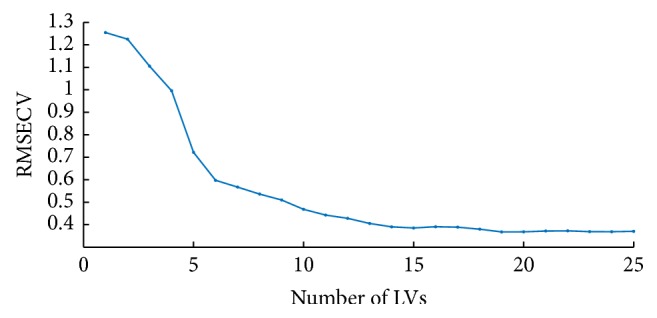
Variation of RMSECV in PLS model with the number of LVs increasing.

**Figure 7:**
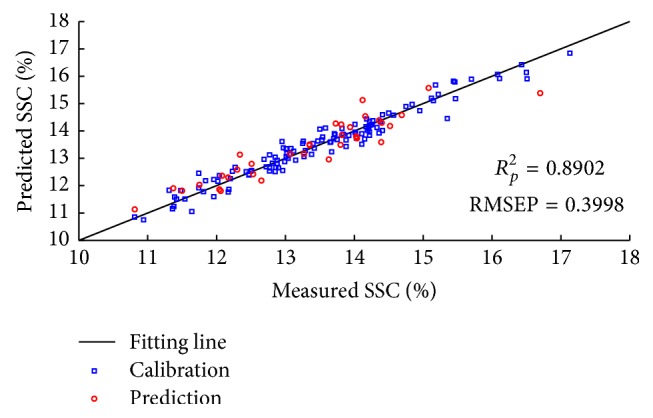
Prediction of soluble solids content (SSC) of apple in PLS regression with the best preprocessing method.

The full-spectrum (4000–10000 cm^−1^) PLS models were developed using raw spectra and preprocessed spectra pretreated by S-G smoothing, MSC, SNV, and MeanN and their combinations, respectively. In order to improve the predictive accuracy and reduce the overfitting problem, the optimal number of latent variables (LVs) was determined by 10-fold cross-validation. As can be seen in Figure 6, the correlation coefficient of cross-validation (RMSECV) showed a descending trend with the increase of LVs and the best range of LVs number is 6–14 because overfitting appears when LVs number is too high. Therefore, the PLS model with no more than 14 LVs was selected to predict the SSC of apples [14].  Table 2 presents the performances of optimal PLS models using these preprocessing methods in SSC prediction, which were determined by RMSEC, RMSECV, RMSEP, and *R*^2^. It can be found that the combination of S-G smoothing and MSC gave better predictions over the other pretreatments with *R*_*P*_^2^ of 0.8902 and RMSEP of 0.3998. Therefore, further analysis was conducted based on the spectra after S-G smoothing and MSC pretreatments. The relationship between the SSC measurements and the predicted values obtained from PLS models is shown in Figure 7.

### 4.4. Models for CARS and RF Selected Spectra

**Figure 8:**
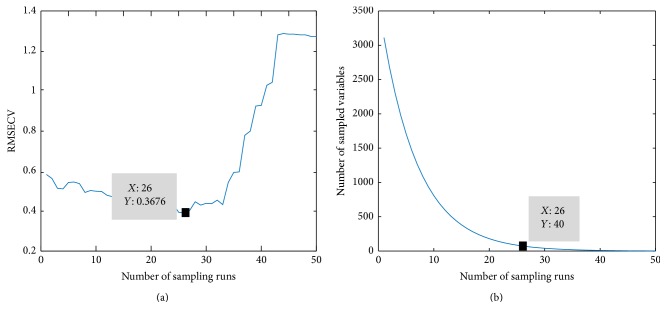
The 10-fold RMSECV values (a) from one CARS running and the changing trend of the number of sampled variables (b) with the increasing of sampling runs.

**Figure 9:**
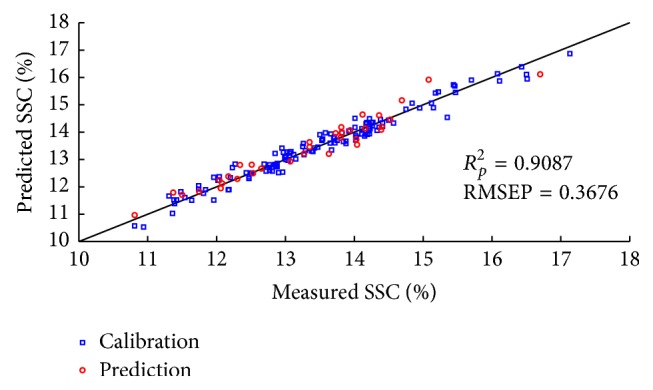
Scatter plots of measured versus predicted values for SSC obtained by the CARS-PLS calibration models.

**Figure 10:**
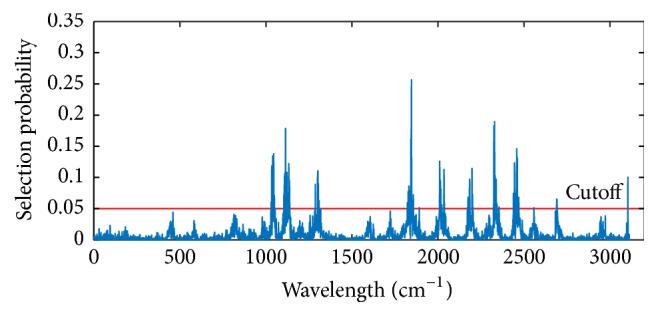
Selection probability of each wavelength for prediction of SSC.

**Figure 11:**
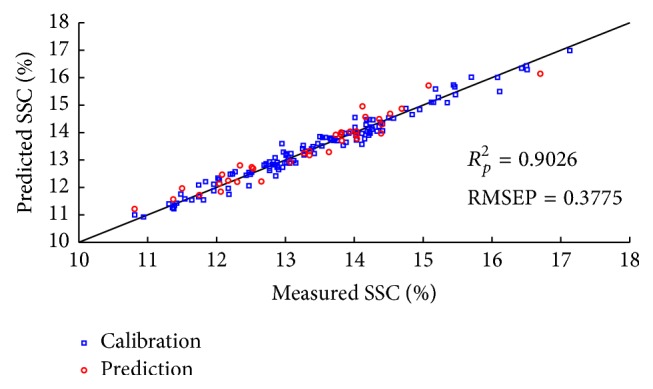
Scatter plots of measured versus predicted values for SSC obtained by the RF-PLS calibration models.

The CARS and RF variable selection methods were used for SSC prediction to pick out effective wavelengths from original data. For each running of CARS in this study, the Monte Carlo sampling runs count was set to 50 and the number of selected variables was determined by 10-fold cross-validation.  Figure 8 gives the 10-fold RMSECV values (Figure 8(a)) and the trend of the number of sampled variables (Figure 8(b)), with the increasing sampling runs from one CARS running. In Figure 8(a), RMSECV values decreased slowly at first because of the elimination of uninformative variables and then increased rapidly with the loss of effective variables. According to the minimal RMSECV value obtained in the 26th sampling run marked by a black square in Figure 8(a), the optimal variable subset was determined, while the corresponding number of sampled variables was 40, which was marked in Figure 8(b). Consequently, 40 variables were selected by CARS as the most effective wavelengths to establish PLS models for apple SSC determination.  Figure 9 shows the predicted values of apple SSC from CARS-PLS models. Furthermore, RF was also carried out to select important spectra variables for comparison with the CARS.  Figure 10 displays the selection probability (SP) of wavelengths determined by RF algorithm; the larger the SP is, the more important the corresponding wavelength is. Set the cutoff threshold of SP in Figure 10 to be 0.05, where wavelengths with SP over cutoff threshold were chosen for further analysis, while others would be eliminated. Therefore, 107 important variables were set as the inputs to develop RF-PLS models and the SSC predictions were illustrated in Figure 11. As can be seen in Table 3, the CARS-PLS and RF-PLS models performed even better than full spectra PLS regression, with RMSEP of 0.9087, 0.9026, and 0.8902, respectively. The results showed that CARS and RF were both effective methods to eliminate useless variables and improve accuracy of SSC prediction for the calibration models by selecting important wavelengths. It is worth mentioning that such significant improvements were achieved using only around 1.2% and 3.4% of variables of full-range spectra, respectively.

## 5. Conclusion

In this research, FT-NIR spectroscopy combined with multivariate analysis and wavelength selection algorithm was used for eliminating uninformative biological variability in “Fuji” apple SSC inspection. Wavelengths ranging from 4000 cm^−1^ to 10000 cm^−1^ were pretreated by four different methods (S-G smoothing, MSC, SNV, and MeanN) as well as their combinations to remove irrelevant information. Then the performance of PLS calibration models based on these preprocessing methods was analyzed and compared. Finally, CARS and RF were used to select the optimal variables for further elimination of apple biological variability. Results showed that the combination of S-G smoothing (39-point) and MSC achieved better preprocessing effect for SSC prediction (*R*_*P*_^2^ = 0.8902; RMSEP = 0.3998). Moreover, CARS-PLS was found to have the optimal performance (*R*_*P*_^2^ = 0.9087; RMSEP = 0.3676) using fewer variables compared with full spectra PLS and RF-PLS models. In conclusion, the satisfactory prediction accuracy indicated that FT-NIR combined with S-G smoothing, MSC, and CARS can be used to eliminate the uninformative biological variability, and it had a potential application for online detection of apple SSC. However, there is no single, universally optimal technique for selecting key wavelengths and preprocessing spectra in a general case. Structured investigation of model robustness (across new growing districts, conditions, seasons, etc.) should be conducted in further research.

## Figures and Tables

**Table 1:** Statistic values of SSC (°Brix) for the calibration and prediction sets of apples.

Data set	Samples	Minimum	Maximum	Mean	SD
Calibration	120	10.81	17.13	13.49	1.37
Prediction	40	10.83	16.70	13.32	1.22

**Table 2:** Prediction results for SSC by PLS with different pretreatments in calibration, validation, and prediction processes.

Pretreatment	LVs	Calibration		Cross-validation		Prediction	
		*R*_*C*_^2^	RMSEC	*R*_CV_^2^	RMSECV	*R*_*P*_^2^	RMSEP
Raw	14	0.9532	0.2764	0.9066	0.3903	0.8720	0.4197
S-G	14	0.9429	0.3053	0.9052	0.3933	0.8863	0.4079
MSC	14	0.9681	0.2282	0.9107	0.3816	0.8429	0.4795
SNV	13	0.9513	0.2817	0.9050	0.3937	0.8834	0.4132
MeanN	13	0.9476	0.2923	0.9081	0.3873	0.8816	0.4163
S-G and MSC	14	0.9478	0.3067	0.9177	0.3565	0.8902	0.3998
S-G and MeanN	14	0.9474	0.3063	0.9089	0.3756	0.8883	0.4044
MSC and MeanN	14	0.9681	0.2282	0.9107	0.3816	0.8429	0.4795
MSC and SNV	13	0.9514	0.2817	0.9050	0.3904	0.8834	0.4131

**Table 3:** The prediction results of apple SSC by different PLS models.

Model	Number of variables	LVs	Calibration		Prediction	
			*R*_*C*_^2^	RMSEC	*R*_*P*_^2^	RMSEP
Full-spectra PLS	3112	14	0.9532	0.2764	0.8902	0.3998
CARS-PLS	40	14	0.9492	0.2878	0.9087	0.3676
RF-PLS	107	13	0.9469	0.2942	0.9026	0.3775

## References

[1] Peng Y., Lu R. (2008). Analysis of spatially resolved hyperspectral scattering images for assessing apple fruit firmness and soluble solids content. *Postharvest Biology and Technology*.

[2] Giovanelli G., Sinelli N., Beghi R., Guidetti R., Casiraghi E. (2014). NIR spectroscopy for the optimization of postharvest apple management. *Postharvest Biology and Technology*.

[3] Peirs A., Lammertyn J., Ooms K., Nicola B. M. (2001). Prediction of the optimal picking date of different apple cultivars by means of VIS/NIR-spectroscopy. *Postharvest Biology and Technology*.

[4] Mendoza F., Lu R. F., Cen H. Y. (2014). Grading of apples based on firmness and soluble solids content using Vis/SWNIR spectroscopy and spectral scattering techniques. *Journal of Food Engineering*.

[5] Lu R. (2004). Multispectral imaging for predicting firmness and soluble solids content of apple fruit. *Postharvest Biology and Technology*.

[6] Peiris K. H. S., Dull G. G., Leffler R. G., Kays S. J. (1999). Spatial variability of soluble solids or dry-matter content within individual fruits, bulbs, or tubers: implications for the development and use of NIR spectrometric techniques. *HortScience*.

[7] Bobelyn E., Serban A.-S., Nicu M., Lammertyn J., Nicolai B. M., Saeys W. (2010). Postharvest quality of apple predicted by NIR-spectroscopy: study of the effect of biological variability on spectra and model performance. *Postharvest Biology and Technology*.

[8] Liu Y., Ying Y. (2005). Use of FT-NIR spectrometry in non-invasive measurements of internal quality of 'Fuji' apples. *Postharvest Biology and Technology*.

[9] Guo Z., Huang W., Peng Y., Chen Q., Ouyang Q., Zhao J. (2016). Color compensation and comparison of shortwave near infrared and long wave near infrared spectroscopy for determination of soluble solids content of 'Fuji' apple. *Postharvest Biology and Technology*.

[10] Peirs A., Scheerlinck N., Nicolaï B. M. (2003). Temperature compensation for near infrared reflectance measurement of apple fruit soluble solids contents. *Postharvest Biology and Technology*.

[11] Fan S. X., Huang W. Q., Guo Z. M., Zhang B. H., Zhao C. J., Qian M. (2015). Assessment of Influence of Origin Variability on Robustness of Near Infrared Models for Soluble Solid Content of Apples. *Chinese Journal of Analytical Chemistry*.

[12] Zhang B., Dai D., Huang J., Zhou J., Gui Q. (2017). Influence of physical and biological variability and solution methods in fruit and vegetable quality non-destructive inspection by using imaging and near-infrared spectroscopy techniques: a review. *Critical Reviews in Food Science and Nutrition*.

[13] Peirs A., Scheerlinck N., Touchant K., Nicolaï B. M. (2002). Comparison of Fourier transform and dispersive near-infrared reflectance spectroscopy for apple quality measurements. *Biosystems Engineering*.

[14] Fan S., Zhang B., Li J., Huang W., Wang C. (2016). Effect of spectrum measurement position variation on the robustness of NIR spectroscopy models for soluble solids content of apple. *Biosystems Engineering*.

[15] Jie D., Xie L., Fu X., Rao X., Ying Y. (2013). Variable selection for partial least squares analysis of soluble solids content in watermelon using near-infrared diffuse transmission technique. *Journal of Food Engineering*.

[16] Lu R., Peng Y. (2005). Assessing peach firmness by multi-spectral scattering. *Journal of Near Infrared Spectroscopy*.

[17] Zhang B., Huang W., Gong L. (2015). Computer vision detection of defective apples using automatic lightness correction and weighted RVM classifier. *Journal of Food Engineering*.

[18] Li J., Huang W., Chen L. (2014). Variable selection in visible and near-infrared spectral analysis for noninvasive determination of soluble solids content of ‘Ya’ pear. *Food Analytical Methods*.

[19] Zou X., Zhao J., Li Y. (2007). Selection of the efficient wavelength regions in FT-NIR spectroscopy for determination of SSC of 'Fuji' apple based on BiPLS and FiPLS models. *Vibrational Spectroscopy*.

[20] Leiva-Valenzuela G. A., Lu R., Aguilera J. M. (2013). Prediction of firmness and soluble solids content of blueberries using hyperspectral reflectance imaging. *Journal of Food Engineering*.

[21] Kennard R. W., Stone L. A. (1969). Computer aided design of experiments. *Technometrics*.

[22] Travers S., Bertelsen M. G., Petersen K. K., Kucheryavskiy S. V. (2014). Predicting pear (cv. Clara Frijs) dry matter and soluble solids content with near infrared spectroscopy. *LWT - Food Science and Technology*.

[23] Li H., Liang Y., Xu Q., Cao D. (2009). Key wavelengths screening using competitive adaptive reweighted sampling method for multivariate calibration. *Analytica Chimica Acta*.

[24] Fan W., Shan Y., Li G., Lv H., Li H., Liang Y. (2012). Application of Competitive Adaptive Reweighted Sampling Method to Determine Effective Wavelengths for Prediction of Total Acid of Vinegar. *Food Analytical Methods*.

[25] Hu M., Dong Q., Liu B., Opara U. L., Chen L. (2015). Estimating blueberry mechanical properties based on random frog selected hyperspectral data. *Postharvest Biology and Technology*.

[26] Li H.-D., Xu Q.-S., Liang Y.-Z. (2012). Random frog: an efficient reversible jump Markov Chain Monte Carlo-like approach for variable selection with applications to gene selection and disease classification. *Analytica Chimica Acta*.

[27] Yun Y.-H., Li H.-D., E. Wood L. R. (2013). An efficient method of wavelength interval selection based on random frog for multivariate spectral calibration. *Spectrochimica Acta Part A: Molecular and Biomolecular Spectroscopy*.

[28] Li J., Huang W., Zhao C., Zhang B. (2013). A comparative study for the quantitative determination of soluble solids content, pH and firmness of pears by Vis/NIR spectroscopy. *Journal of Food Engineering*.

